# Disproportionately high failure to rescue rates after resection for colorectal cancer in the geriatric patient population – A nationwide study

**DOI:** 10.1002/cam4.4784

**Published:** 2022-04-27

**Authors:** Johannes Diers, Philip Baum, Kai Lehmann, Konstatin Uttinger, Nikolas Baumann, Sebastian Pietryga, Mohammed Hankir, Niels Matthes, Johann F. Lock, Christoph‐Thomas Germer, Armin Wiegering

**Affiliations:** ^1^ Department of General, Visceral, Transplant, Vascular and Paediatric Surgery, University Hospital University of Würzburg Würzburg Germany; ^2^ Department of Thoracic Surgery Thoraxklinik at Heidelberg University Hospital Heidelberg Germany; ^3^ Department of General, Visceral and Vascular Surgery Charité University Hospital Berlin Campus Benjamin Franklin Berlin Germany; ^4^ Comprehensive Cancer Centre Mainfranken University of Würzburg Medical Centre Würzburg Germany; ^5^ Department of Biochemistry and Molecular Biology University of Würzburg Würzburg Germany

**Keywords:** colorectal cancer, geriatric, octogenerians, surgery

## Abstract

**Background:**

Colorectal cancer incidence increases with patient age. The aim of this study was to assess, at the nationwide level, in‐hospital mortality, and failure to rescue in geriatric patients (≥ 80 years old) with colorectal cancer arising from postoperative complications.

**Methods:**

All patients receiving surgery for colorectal cancer in Germany between 2012 and 2018 were identified in a nationwide database. Association between age and in‐hospital mortality following surgery and failure to rescue, defined as death after complication, were determined in univariate and multivariate analyses.

**Results:**

Three lakh twenty‐eight thousands two hundred and ninety patients with colorectal cancer were included of whom 77,287 were 80 years or older. With increasing age, a significant relative increase in right hemicolectomy was observed. In general, these patients had more comorbid conditions and higher frailty. In‐hospital mortality following colorectal cancer surgery was 4.9% but geriatric patients displayed a significantly higher postoperative in‐hospital mortality of 10.6%. The overall postoperative complication rate as well as failure to rescue increased with age. In contrast, surgical site infection (SSI) and anastomotic leakage (AL) did not increase in geriatric patients, whereas the associated mortality increased disproportionately (13.3% for SSI and 29.9% mortality for patients with AI, both *p* < 0.001). Logistic regression analysis adjusting for confounders showed that geriatric patients had almost five‐times higher odds for death after surgery than the baseline age group below 60 (OR 4.86; 95%CI [4.45–5.53], *p* < 0.001).

**Conclusion:**

Geriatric patients have higher mortality after colorectal cancer surgery. This may be partly due to higher frailty and disproportionately higher rates of failure to rescue arising from postoperative complications.

## Graphical abstract

Patients above the age of 80 years show a disproportional high mortality and failure to rescue after resection of colorectal cancer.

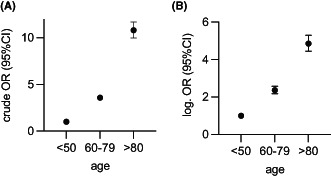

## 1 INTRODUCTION

The incidence of colorectal cancer increases with age.^1^ Since there has been a general increase in life expectancy globally in recent decades, the number of colorectal cancer cases in the elderly population is expected to rise significantly (https://www.cdc.gov/nchs/fastats/life‐expectancy.htm). Geriatric patients above 80 years (octogenarians) and 90 years (nonagenarians) form a unique group of patients, with an often reduced functional as well as cognitive capacity, increased number of co‐morbidities, and a higher fragility.^2^ Despite this, elderly patients are often excluded from clinical trials and large cohort analyses leading to the fact that geriatric patients have been severely underrepresented in the evaluation of clinical treatment.^3^ Notably, while elderly patients often have more comorbidities and suffer from increased frailty, surgical resection remains their only curative modality for CRC.^4^ Several previous single‐ and multi‐hospital studies with relatively low numbers of included patients have demonstrated that elderly patients are at a higher risk for postoperative complications and mortality.^5^, ^6^, ^7^, ^8^ Large nationwide studies assessing the effects of age and comorbidity on morbidity and mortality in the elderly after resection of colorectal cancer are lacking.

The aim of this analysis was to assess the influence of advanced age (above 80) on the kind of surgical treatment, occurrence of complications, failure to rescue, and mortality in patients undergoing colon or rectal resections for colorectal carcinoma (CRC). Nationwide data from 2012 to 2018 for patients with a diagnosis of either colon (C18), recto sigmoidal (C19), or rectal (C20) cancer and a simultaneous therapy code for a colorectal resection were included and analyzed. The primary endpoint was the in‐hospital mortality rate according to patient age. Secondary endpoints were the occurrence of complications and the associated “failure to rescue” in patients as well as the impact of hospital volume on age‐related outcomes.

## 2 METHODS

For this retrospective cohort study, we used anonymized individual inpatient billing data from the nationwide German diagnosis‐related groups (DRG) registry accessed via the Research Data Centre of the Federal Statistical Office by controlled remote data analysis. No other data source was used. All inpatients with a DRG‐code C18, C19, and C20 for colon, rectosigmoid, and rectal cancer, respectively, as the main diagnosis who underwent colon or rectum resection in Germany between January 01, 2012 and December 31, 2018 were identified (Table S1 for procedure codes). Both elective and emergency admissions in patients ≥18 years were considered. Procedures were approached hierarchically within each patient and the more radical intervention was designated the principal intervention to avoid double counting of interventions. Hospital caseload was computed for each year and hospitals were divided into five volume quintile categories of approximately equal patient numbers according to their yearly number of colon and rectum resections as a proxy variable for surgical expertise. Hospital allocation to volume quintiles could vary between years. German inpatient billing data include anonymized unique patient and hospital identifiers, DRG codes of primary and secondary diagnoses, concomitant procedure codes, patient sex, age, duration of mechanical ventilation, mass transfusion, and length of stay (LOS). The German adaptation of the ICD‐10‐GM codes and the German procedure (OPS) codes in the relevant versions were used for this study [https://www.dimdi.de/static/de/klassi/icd‐10‐gm/kodesuche/vorgaenger.htm; accessed July 18, 2020]. For each patient, a three category‐frailty score (low, intermediate, and high risk) based on the secondary diagnoses was computed as described elsewhere.^9^, ^10^ Moreover, we computed a comorbidity score for each patient as described by Stausberg et al. to account for variation in the comorbidity profile of patients.^11^ Patients were categorized into three age groups with the third age group comprising the geriatric population (patient age < 60, 60–79, and ≥ 80 years). Data were cross tabulated and crude associations between categorical variables were evaluated using chi‐squared tests. Trends were assessed with a nonparametric test for trend.^12^ Crude odds ratios (OR) between in‐hospital mortality, failure to rescue (FtR, defined as in‐hospital death following a complication) hospital volume quintile and patient age group, the main independent variable, as well as with other secondary independent variables (sex, comorbidity, etc.) were calculated to identify potential confounders. Mantel–Haenszel method was used to screen for relevant effect modification. We determined the correlation between the different pairs of candidate variables for multiple regression analysis to detect multicollinearity. We estimated the effect of patient age on in‐hospital mortality and on FtR by multivariable logistic regression analysis accounting for patient clustering within hospitals by means of hospital as a random effect. The accuracy of the random‐effects estimators of the multivariable models was checked by refitting the models for different numbers of quadrature points and subsequent comparison of the values of the estimators. 10^−4^ was the maximum acceptable relative difference between the quadrature points. Model performance was assessed using likelihood ratio tests.

Statistical analyses were performed with Stata 14.2 (StataCorp LP, College Station, Texas, USA). A *p*‐value ≤0.05 was considered significant and 95% confidence intervals were reported wherever possible. The authors followed RECORD Guideline for good practice of secondary data analysis.^13^

## 3 RESULTS

A total of 330,043 individual datasets of inpatients ≥18 years with the diagnoses of colon or rectal cancer (ICD‐10‐GM codes C18, C19, and C20) and a procedure code indicating colon or rectal resection between January 01, 2012 and December 31, 2018 were identified in the German DRG‐registry. Of these 1753 [0.5%] patients were excluded due to missing data, leaving 328,290 datasets for final analysis. Median age was 72 years and 145,968 (44.5%) were female. Roughly 1/5 were younger than 60 years, 3/5 between 60 and 80 years, and 1/5 were above 80 years of age (*n* = 77,287, 23.5%). Two thirds had colon cancer (67.4%, *n* = 221,179) and one third had rectal cancer (*n* = 107,111). The most frequent cancer location and subsequent procedures were right colon cancer with right hemicolectomy (*n* = 106,223; 32.4%), sphincter preserving low anterior resection (*n* = 49,470; 15.1%) and sigmoid resection (*n* = 34,628; 10.6%) (Table 1). The fraction of patients treated for colon cancer steadily increased across age groups and peaked in above 80 years (35,209, 58.0% in the <60 years vs. 59,593, 77.1% >80 years, *p* < 0.001). When stratifying cancer location according to patient age, a clear shift to the right‐sided colon was observed with increasing age (right hemicolectomy: 13213, 21.8% vs. 32,896, 42.6% *p* < 0.001) (Table S2). The relative proportion of non‐restorative colectomy (Hartmann's procedure) for left‐sided and rectal cancer increased with patient age. In above 80's, it accounted for 16.0% (*n* = 3204) and in the youngest patients it accounted for 7.7% (*n* = 1272) of all left‐sided interventions (Table 1). Laparoscopic access decreased with age (*n* = 21,052; 34.7% in 60 years vs. *n* = 14,607; 18.9% in above 80's, *p* < 0.001). In addition, the relative percentage of emergency surgeries increased with age (*n* = 13,371; 18.1% vs. *n* = 26,952, 25,9%; *p* < 0.001) (Table 1).

**TABLE 1:** Characteristics of patients undergoing colorectal cancer resection 2012–2018

	Age group				
	<60	60–79	≥80	Total	*P*
Total no. of patients (% of total patient *n*°)	60,665 (18.5)	190,338 (58.0)	77,287 (23.5)	328,290 (100)	
In‐hospital deaths (%)	656 (1.1)	7125 (3.8)	8166 (10.6)	15,997 (4.9)	<0.001^a^
No. of women (%)	25,377 (41.8)	77,885 (40.9)	42,706 (55.3)	145,968 (44.5)	<0.001
Length of hospital stay (days, median and sd)	13 (±12.1)	15 (±14.0)	20 (±13.5)	16 (±13.8)	<0.001^a^
Cancer location					
Colon (% of total patient *n*°)	35,209 (58.0)	126,377 (57.1)	59,593 (77.1)	221,179 (67.4)	<0.001
Mortality	446 (1.3)	5188 (4.1)	6520 (10.9)	12,154 (5.5)	<0.001
Rectum (% of total patient *n*°)	25,456 (42.0)	63,961 (33.6)	17,694 (22.9)	107,111 (32.6)	<0.001
Mortality	210 (0.8)	1987 (3.1)	1646 (9.3)	3843 (3.6)	<0.001
Surgical access					
Laparoscopic	21,052 (34.7)	49,827 (26.2)	14,607 (18.9)	85,486 (26.0)	<0.001
Mortality	80 (0.4)	829 (1.7)	812 (5.6)	1721 (2.0)	0.003
Hartmann procedure (% of left‐sided interventions)	1272 (7.7)	5061 (10.3)	3204 (16.0)	9537 (11.1)	<0.001
Mortality	79 (6.2)	715 (14.1)	803 (25.1)	1597 (14.3)	<0.001
Emergency admission	13,371 (18.1)	45,757 (19.4)	26,952 (25.9)	86,080 (26.2)	<0.001
Mortality	280 (2.1)	2905 (6.4)	3934 (14.6)	7119 (8.3)	<0.001^a^
Frailty score (mean, sd)	0.2 (±0.4)	0.3 (±0.6)	0.6 (±0.7)	0.4 (±0.6)	<0.001^a^
0 (% of within age group)	49,879 (82.2)	133,804 (70.3)	38,534 (49.9)	222,217 (67.7)	<0.001
Mortality	165 (0.3)	1740 (1.3)	1848 (4.8)	3753 (1.7)	<0.001
1 (% of within age group)	10,025 (16.5)	48,985 (25.7)	31,459 (40.7)	90,469 (27.6)	<0.001
Mortality	380 (3.8)	3986 (8.1)	4617 (14.7)	8983 (9.9)	<0.001
2 (% of within age group)	761 (1.3)	7549 (4.0)	7294 (9.4)	15,604 (4.8)	<0.001
Mortality	111 (14.6)	1449 (19.2)	1701 (23.3)	3261 (20.9)	<0.001

*Note*: X due to data protection legislation no data provided.

Abbreviation: md, missing data.

^a^ Nonparametric test for trend, if no indication chi‐squared test.

The mean frailty score steadily rose from 0.2 to 0.6 (*p* < 0.001 for trend) with increasing age group. The same held true of the mean comorbidity score (*p* < 0.001 for trend) as reflected by specific comorbidities like congestive heart failure (*n* = 1081/60,665 vs. *n* = 17,442/77,287 or 1.8% vs. 22.6%, *p* < 0.001), cardiac arrhythmias (*n* = 1358/60,665 vs. *n* = 24,331/77,287 or 2.2% vs. 31.5%, *p* < 0.001), and chronic kidney failure (*n* = 1463/60,665 vs. *n* = 19,163/77,287, or 2.4% vs. 24.8%, *p* < 0.001) (Table S2). Interestingly, certain comorbidities displayed slightly different patterns. For instance, obesity peaked in 60–79‐year‐olds (*n* = 20,394 or 10.7%) and decreased in above 80 (*n* = 4658; or 6.0%). There was a strong increase in the prevalence of diabetes between the youngest and the middle age group (*n* = 5152 vs. *n* = 44,188, or 8.5% vs. 23.2%), but the subsequent increase in the oldest population was moderate (*n* = 19,339 or 25.0%) (Table S2).

Nationwide in‐hospital mortality following colorectal cancer surgery was 4.9% (*n* = 15,997). Mortality steadily increased across the age groups from 1.1% (*n* = 656) in patients <60 years old to 3.8% (*n* = 7125) in 60–79‐year‐old and peaked in above 80 years with a 10.6% (*n* = 8166). When analyzing individual cancer locations as well as procedures, mortality steadily increased with age (Table 1). In general, mortality was highest for total colectomy (*n* = 485; 11.9%) with an in‐hospital mortality reaching 24.9% for the oldest population (*n* = 199). Followed by subtotal colectomy (*n* = 1656; 6.5%) and rectosigmoid resection (*n* = 20,682; 6.3%) for which above 80´s were also particularly at risk for in‐hospital death (*n* = 803; 13.0% subtotal colonic resection; *n* = 399; 9.9% rectosigmoid resection) (Table S2).

Mortality was, in addition, associated with emergency surgery as well as patient frailty. Patients in the high‐frailty category carried a 20.9% risk of in‐hospital death (*n* = 3261 of 15,604, of which 1701 or 52.2% were ≥ 80 years old) as compared to 9.9% and 1.7% in the intermediate and low‐risk frailty‐categories (*n* = 8983 of 90,469 and 3753 of 222,217 respectively, *p* < 0.001). Patient mortality in the high‐risk group ranged from between 14.6% and 23.3% across age groups and increased with age (Table 1).

Both the cumulative incidence of complications and the failure to rescue rose continuously with increasing patient age. Interestingly, while the occurrence of medical complications increased from 6.8% in those <60 years old and to 21.6% in 80 years or older (*p* < 0.001), the percentage of surgical complications increased less from 21.2% in those <60 years old to 26.8% in above 80 years (*p* < 0.001). While the FtR was 4.1% in those <60 years old after one or more complications were encoded (*n* = 591/14,309), 23.7% of above 80's suffered in‐hospital death after a complication (*n* = 6790/28,631) (Table S3).

General non‐surgical complications investigated included for example pulmonary embolism and acute kidney injury (AKI). The incidence of the former increased from 0.8% below 60 to 1,4% in the above 80 years, while the associated mortality dramatically increased from 12.3% to 28.9% (*n* = 307/1062, *p* < 0.001 for trend). The latter displayed a continuous rise across all age groups reaching a 9.9% prevalence in those ≥80 years old (*n* = 7628/77,287). FtR after AKI was seen in more than 1/3 of all patients in this age group (39.8%, *n* = 3032/7628) (Table S3).

**FIGURE 1:**
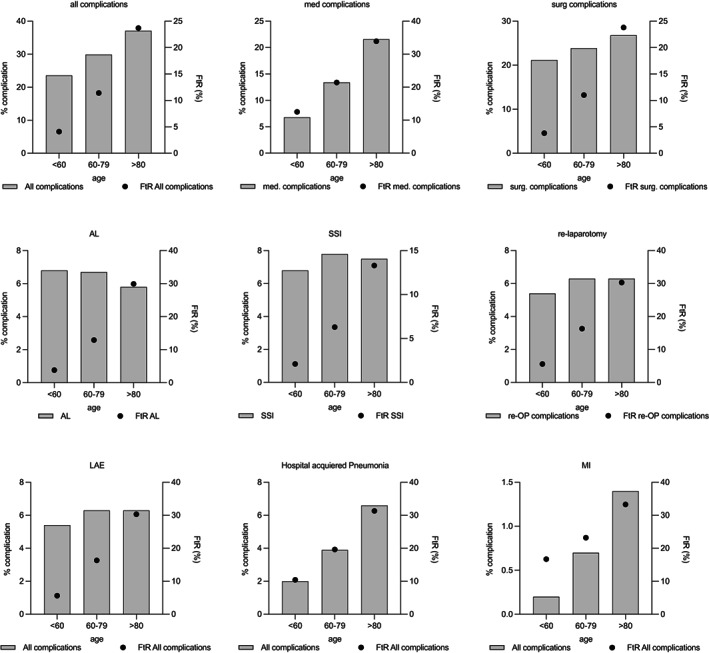
Failure to rescue according to patients age. Gray bar: percentage of complications, black dot: FtR according to age. (*P*‐values for all associations *p* < 0.001 [see Table S2])

While the occurrence of surgical complications only moderately increased with increasing age, mortality rapidly increased (3.8% FtR in those <60 years old vs. 23.8% FtR in geriatric patients, *p* < 0.001) (Table 2). Surgical complications investigated included among others anastomotic leakage (AL) and surgical site infection (SSI). SSI was seen in between 6.0% and 7.8% of all cases peaking in 60–79 years old and decreasing thereafter. The FtR associated with SSI, however, continuously rose across all age groups, and culminated at 13.3% in geriatric patients (*n* = 769/5769; *p* < 0.001 for trend). Surprisingly occurrence of AL decreased slightly with increasing age from 6.8% of all resections requiring the formation of an anastomosis in those <60 years old (*n* = 4102) to 5.8% in those ≥80 years old (*n* = 4550). However, FtR steadily increased with age and was as high as 29.9% in geriatric patients (*n* = 1361) (Table S3; Figure 1).

**TABLE 2:** Characteristics of patients undergoing colorectal cancer resection 2012–2018, according to hospital volume quintile

	Hospital volume quintile						
	Very low	Low	Medium	High	Very high	Total	*P*
Mean no. of hospitals/year	459	205	142	108	70	985	n/a
Mean no. of patients/year	9157	9423	9360	9612	9607	47,189	n/a
No of patients	63,772	65,607	65,168	66,865	66,878	328,290	n/a
In‐hospital deaths	4008 (6.3)	3569 (5.4)	3040 (4.7)	2832 (4.2)	2548 (3.8)	15,997 (4.9)	<0.001^a^
Median patient age (sd)	74 (±11.5)	73 (±11.6)	72 (±11.8)	72 (±11.8)	71 (±12.2)	72 (±11.8)	<0.001^a^
Age group							
<60 years (% of quintil)	9588 (15.8)	11,136 (18.4)	12,106 (20.0)	13,210 (21.8)	14,625 (24.1)	60,665 (18.5)	<0.001
Mortality	133 (1.4)	154 (1.4)	98 (0.8)	138 (1.0)	133 (0.9)	656 (1.1)	<0.001
60–79 years (% of quintil)	35,971 (18.9)	37,954 (19.9)	38,040 (20.0)	39,400 (20.7)	38,973 (20.5)	190,338 (58.0)	<0.001
Mortality	1672 (4.7)	1557 (4.1)	1414 (3.7)	1324 (3.4)	1208 (3.1)	7175 (3.6)	<0.001
≥ 80 years (% of quintil)	18,213 (23.6)	16,517 (21.4)	15,022 (19.4)	14,255 (18.4)	13,280 (17.2)	77,287 (23.5)	<0.001
Mortality	2203 (12.1)	1858 (11.3)	1528 (10.2)	1370 (9.6)	1207 (9.1)	8166 (9.6)	<0.001
Frailty score (mean, sd)	0.42 (±0.6)	0.39 (±0.6)	0.37 (±0.6)	0.35 (±0.6)	0.32 (±0.5)	0.37 (±0.6)	<0.001^a^
Comorbidity score (mean, sd)	102.8 (±5.7)	102.4 (±5.6)	102.1 (±5.5)	102.0 (±5.4)	101.9 (±5.3)	102.2 (±5.5)	<0.001a^a^

Abbreviation: n/a, not applicable.

a Nonparametric test for trend.

German hospitals performing colorectal cancer resections were next divided into five caseload quintiles of equal size according to the annual number of colorectal interventions. On average, 65,658 patients were treated in each volume quintile during the observation period. Colorectal resections in the lowest caseload quintile were 20 patients per year in 459 hospitals, whereas 137 patients were operated each year in the highest quintile volume with 70 hospitals (Table 2). Median patient age decreased from 74 to 71 years from the lowest to the highest volume quintile (*p* < 0.001 for trend). In‐house mortality across all age groups also steadily decreased from 6.3% to 3.8% (*p* < 0.001 for trend) as did the mean frailty score (0.42 to 0.32; *p* for trend <0.001). Geriatric patients were more likely to be treated in low‐volume centers. For instance, 23.6% of all patients treated in very‐low volume hospital were 80 years or older, whereas it was 17.2% of all patients in the hospitals performing the most resections belonged to this age group. Mortality in geriatric patients, however, was significantly lower in high volume centers with 9.1% in‐hospital mortality in hospitals of the highest volume category (*n* = 1207) versus 12.1% (*n* = 2203) in hospitals performing the least colorectal resections (*p* < 0.001) (Table 2).

**FIGURE 2:**
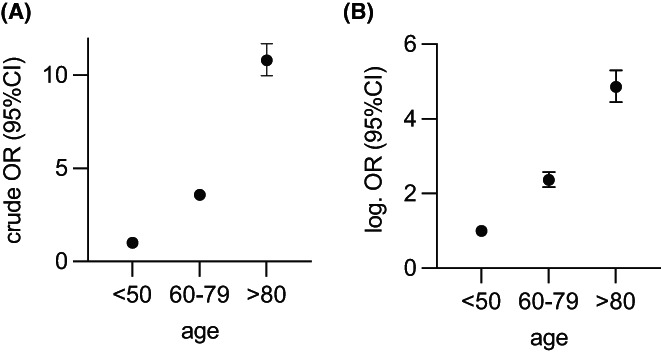
(A) Unadjusted in‐house mortality and 95% CI; (B) adjusted in‐house mortality and 95% CI according to patients age group (*P*‐values for all associations *p* < 0.001 [see Table 3])

The crude ORs for in‐hospital mortality after CRC‐resection increased to 3.58 [95% CI 3.31–3.88] in the middle age group. In patients above 80 years, it was more than 10‐fold the baseline OR constituted by patients <60 years old (OR 10.81 [95% CI 9.97–11.71], *p* < 0.001 for both age groups compared to the baseline) (Table 3). Logistic regression analysis adjusting for sex, patient frailty, tumor location, and hospital caseload demonstrated that increasing age continued to predict significantly worse outcomes. Patients aged 60–79 had 2.6 times higher whereas geriatric patients had a nearly 5‐times higher risk for in‐house mortality (for the ≥80 years old OR 4.86; 95%CI [4.45–5.53], *p* < 0.001) (Table 3, Figure 2). Both in univariate and multivariate analyses, increasing age was significantly associated with increased FtR after occurrence of any complication. When adjusting for the effects of sex, patient comorbidity, hospital case load, cancer location and type of admission, age remained an independent risk factor for increased failure to rescue. The odds of dying after suffering a complication approximately doubled with each age group (OR 2.23 [2.03–2.45] for 60–79 years and 4.11 [3.75–4.12] for patients above 80 years, both *p* < 0.001) (Tables S4 and S5).

**TABLE 3:** Univariate and multivariate analysis for in hospital mortality of patients undergoing colorectal cancer resection 2012–2018

	Crude odds ratios to determine factors influencing in‐house mortality		Logistic regression analysis of in‐hospital mortality by age, including hospital as random effect	
	Crude odds ratio	*P*	Adjusted odds ratio [95% CI]	*P*
Age				
<60 years	1.0		1.0	
50–79 years	3.58 [3.31–3.88]	<0.001	2.37 [2.17–2.58]	<0.001
≥80 years	10.81 [9.97–11.71]	<0.001	4.86 [4.45–5.30]	<0.001
Sex				
F	1.0		1.0	
M	1.18 [1.14–1.22]	<0.001	1.08 [1.04–1.12]	<0.001
Comorbidity			1.25 [1.25–1.25]	<0.001
Caseload quintile			0.94 [0.92–0.96]	<0.001
Very low	1.0			
Low	0.86 [0.82–0.90]	<0.001		
Medium	0.73 [0.70–0.77]	<0.001		
High	0.66 [0.63–0.69]	<0.001		
Very high	0.59 [0.56–0.62]	<0.001		
Location				
Colon cancer	1.0		1.0	
Rectum cancer	0.64 [0.62–0.66]	<0.001	0.89 [0.85–0.92]	<0.001
Admission				
Elective	1			
Emergency	2.37 [2.29–2.45]	<0.001	1.48 [1.43–1.54]	<0.001

## 4 DISCUSSION

In this study, the influence of aging on in‐house mortality after resection of CRC was investigated using nationwide German DRG data. An increase in mortality with patient age was found to be independent of comorbidities and frailty. While, as expected, the number of comorbidities and postoperative complications increased with patient age, a disproportionate increase in FtR with age was surprisingly found.

Although geriatric patients represent about 20% of all patients with CRC, they are largely not represented in clinical studies. It is therefore imperative to analyze this group of patients to broaden databases. With more than 77,000 patients over 80 years of age analyzed, this is to our knowledge the most comprehensive study to date. Our study confirms a recent publication which found that right‐sided colon cancer is more common in the elderly population.^14^ In line with previous work with fewer patients, an increase in complications with age was found. However, the decisive factor was found not to be the increase in complications, but rather the disproportionately high mortality when a complication occurred. Thus, in patients under 60 years of age, one or more complications occurred in 23.6% with a mortality rate of 4.1%. In contrast, the overall rate of complications in patients over 80 years of age increased by a factor of 1.6, whereas the FtR increased 5.8‐fold with a mortality rate of 23.7% in geriatric patients suffering from one or more postoperative complications. When analyzing‐specific surgical complications like SSI and anastomotic leakage, they decreased with age. For anastomotic leakage, this is probably due to a preselection of patients receiving an anastomosis and not of high clinical relevance. In contrast to this decrease, however, lethality increased disproportionately in the case of anastomotic leakage. This finding has a direct impact on the counseling and care of patients over 80 years of age. The disproportionately high mortality in the event of a complication must be addressed in the course of preoperative patient information and, if necessary, a protective stoma must be applied.

In addition to age and the presence of comorbidities, there is increased frailty, especially in older patients, which often does not correlate with age and the presence of concomitant diseases. To represent this elementary factor, we used a frailty score validated on DRG data. The score showed a significant correlation with age and was associated with increased mortality supporting the argument that besides age, the individual performance status, especially in younger patients needs to be considered.

Taking the disproportional increase of mortality in older and more frailer patients in case of complication into account the prevention of complications seems to be the key‐factor for improved outcome. In recent years the concept of enhanced recovery after surgery and specific geriatric intervention has been proven to be beneficial. For example, a meta‐analysis of 16 RCT has shown that while the occurrence of surgical complications did not change the relative risk for non‐surgical complication drops to 0.4.^15^ In the same line‐specific geriatric assessment improves perioperative outcome and outcome during chemotherapy.^16^, ^17^ Considering these, especially older patients would benefit from reduced perioperative complication rate. In the light of these data, it seems striking that patients above 80 years were more likely to be treated in lower volume centers than younger patients. Since geriatric patients are also more often admitted by the emergency department, we hypothesize that older patients are less likely to deliberately choose the treating hospital and may just be admitted to a nearby facility irrespective of its operative performance. In our view further enhancing nationwide board certification programs could improve the access of geriatric patients to high quality surgical care.^18^, ^19^

In line with our data several other studies have also shown that complication and mortality increase with patient's age.^20^, ^21^ A review of the US nationwide inpatient database by Jafari from 2001 until 2010 also demonstrated that morbidity and mortality increase with patient's age. In addition, occurrence of postoperative complications also is the major prognostic factor for long‐term survival, which is even stronger than the tumor stage.^22^

A number of studies have shown that postoperative lethality correlates with the expertise of the hospital.^18^, ^23^, ^24^, ^25^, ^26^ We found that the frequency of complications did not differ depending on the number of cases, but that the FTR decreased significantly with increasing expertise.^18^ It therefore came as a surprise that, in percentage terms, older patients were treated in hospitals with a low number of cases, in which care in the context of complications is usually associated with higher lethality.

This study has some limitations. For example, the data analysis referred only to the inpatient stay of the surgery. Data on readmission and long‐term survival (five‐year survival) were not represented. In this context, it has previously been shown on a relatively low number of patients, that the occurrence of complications dramatically reduces long‐term survival––particularly in geriatric patients.^6^, ^22^, ^27^, ^28^ Additionally, the database we used lacks information on tumor stage as well as the extent of comorbidity and assessment of ASA score and ECOC performance status.

A notable strength of this study is that a large number of patients were mapped over a relevant time period with a low percentage of missing data. In addition, the data collected and submitted for billing was externally audited by the Medical Service of the Health Insurance Funds providing further validation.^29^ A further strength is that the selected endpoint, in house lethality, is clearly defined and subject to low bias.

## 5 CONCLUSION

Patient age represents an independent risk factor in the setting of colorectal cancer resection. This is mainly due to the significantly increased lethality in the context of complications in older patients. Thus, avoidance of complications and improving the failure to rescue rate in octogenarians and nonagenarians is the key for a positive outcome.

## AUTHOR CONTRIBUTIONS

All authors have made substantial contributions to conception and design, or acquisition of data, or analysis and interpretation of data. All authors have been involved in drafting the manuscript or revising it critically for important intellectual content. All authors gave final approval of the version to be published. Each author have participated sufficiently in the work to take public responsibility for appropriate portions of the content. All authors have agreed to be accountable for all aspects of the work in ensuring that questions related to the accuracy or integrity of any part of the work are appropriately investigated and resolved.

## CONFLICT OF INTEREST

The authors declare no conflict of interest.

## ETHICS APPROVAL STATEMENT

Due to complete anonymity, no approval from Würzburg University's ethics committee was required.

## PATIENT CONSENT STATEMENT

N/A.

## PERMISSION TO REPRODUCE MATERIAL FROM OTHER SOURCES

N/A.

## CLINICAL TRIAL REGISTRATION

N/A.

## Supporting information


Table S1‐S5

## Data Availability

Data sharing is not applicable to this article as no new data were created or analyzed in this study.
