# Supplementary material for: Disproportionately high failure to rescue rates after resection for colorectal cancer in the geriatric patient population – A nationwide study
Source: Cancer Med. 2022 Apr 27;11(22):4256–64. doi: 10.1002/cam4.4784 (PMC9678084; doi:10.1002/cam4.4784)
Supplement: Supplementary file 1 — Table S1‐S5 [file CAM4-11-4256-s001.docx]

**Supplementary material**

| **OPS codes** | **ICD-10 GM codes comorbidities** |
| --- | --- |
| Rectosigmoid resection: 5484  Sigmoid resection: 54557, 54585  Left hemicolectomy: 54556, 54581  Transverse resection: 54555  Right hemicolectomy: 54554, 54580  Subtotal colectomy: 54558, 54559, 5455a-d, 54582  Colectomy: 5456, 54583, 54584  Other colonic resections: 54552, 54553  Non‐sphincter‐preserving rectal resection: 5485  Sphincter‐preserving resection and perianal anastomosis: 54846  Sphincter‐preserving low anterior resection: 54845  Sphincter‐preserving anterior resection: 54843, 54844  Other rectal resections: 54556, 54581, 54557, 54585 | Congestive heart failure: I50, I110, I130, I132, I420, I426-7, I429  Arrhythmias: I442, I48, Z450, Z950  Chronic ischemic heart failure: I25  Hypertension: I10, I119, I129, I139, I15  Chronic pulmonary disease: J41, J42, J44, J45, J47  Chronic kidney injury: I129, I131-2, N03-5, N07-8, N11-2, N14-6, N18-9, Z992  Diabetes mellitus: E10-4  Adipositas: E66  Cachexia/malnutrition: R64, R634, E43-4  Dementia: F00-3 |

Supp. Table 1: OPS and ICD-10 GM codes

| **Supplementary table 2 Additional patient characteristics** | | | | | |
| --- | --- | --- | --- | --- | --- |
|  | **Age group** | | | | |
|  | **<60** | **60-79** | **≥80** | **total** | ***P*‡** |
| **Type of surgery** |  |  |  |  |  |
| Colectomy | 1136 (1.9) | 2156 (1.1) | 800 (1.0) | 4092 (1.3) | <0.001 |
| Mortality | 29 (2.6) | 257 (11.9) | 199 (24.9) | 485 (11.9) | <0.001 |
| Subtotal colectomy | 4210 (6.9) | 15033 (7.9) | 6198 (8.0) | 25441 (7.8) | <0.001 |
| Mortality | 60 (1.4) | 793 (5.3) | 803 (13.0) | 1656 (6.5) | <0.001 |
| Rectosigmoid resection | 4349 (7.2) | 12291 (6.5) | 4042 (5.2) | 20682 (6.3) | <0.001 |
| Mortality | 44 (1.0) | 434 (3.5) | 399 (9.9) | 877 (5.5) | <0.001 |
| Right hemicolectomy | 13213 (21.8) | 60114 (31.6) | 32896 (42.6) | 106223 (32.4) | <0.001 |
| Mortality | 190 (1.4) | 2295 (3.8) | 3241 (9.9) | 5726 (5.4) | <0.001 |
| Transverse resection | 925 (1.5) | 3925 (2.1) | 2111 (2.7) | 6961 (2.1) | <0.001 |
| Mortality | 11 (1.2) | 192 (4.9) | 257 (12.2) | 460 (6.6) | <0.001 |
| Left hemicolectomy | 4034 (6.7) | 11966 (6.3) | 4803 (6.2) | 20803 (6.3) | <0.001 |
| Mortality | 38 (0.9) | 489 (4.1) | 580 (12.1) | 1107 (5.3) | <0.001 |
| Sigmoid resection | 6835 (11.3) | 19870 (10.4) | 7923 (10.25) | 34628 (10.6) | <0.001 |
| Mortality | 72 (1.1) | 709 (3.6) | 917 (11.6) | 1698 (4.9) | <0.001 |
| Other colonic resections | 682 (1.1) | 1578 (0.8) | 1063 (1.4) | 3323 (1.0) | <0.001 |
| Mortality | 8 (1.2) | 82 (5.2) | 166 (15.6) | 256 (4.9) | <0.001 |
| Non-sphincter-preserving rectal resection | 4589 (7.6) | 13690 (7.2) | 3863 (5.0) | 22142 (6.7) | <0.001 |
| Mortality | 38 (0.8) | 443 (3.2) | 348 (9.0) | 829 (3.7) | <0.001 |
| Sphincter-preserving resection and perianal anastomosis | 2601 (4.3) | 4851 (2.6) | 674 (0.9) | 8126 (2,5) | <0.001 |
| Mortality | 16 (0.6) | 95 (2.0) | 53 (7.9) | 1635 (3.3) | <0.001 |
| Sphincter-preserving low anterior resection | 12451 (20.5) | 29440 (15.5) | 7579 (9.8) | 49470 (15.1) | <0.001 |
| Mortality | 102 (0.8) | 871 (3.0) | 662 (8.7) | 1635 (3.3) | <0.001 |
| Sphincter-preserving anterior resection | 5640 (9.3) | 15424 (8.1) | 5335 (6.9) | 26399 (8.0) | <0.001 |
| Mortality | 48 (0.9) | 515 (3.3) | 541 (10.1) | 1104 (4.2) | <0.001 |
| **Comorbidities** |  |  |  |  |  |
| Comorbidty score (mean, sd) | 100.9 (±4.2) | 101.9 (±5.4) | 104.2 (±6.2) | 102.2 (±5.5) | <0.001+ |
| Congestive heart failure (% of age group) | 1081 (1.8) | 17514 (9.2) | 17442 (22.6) | 36037 (11.0) | <0.001 |
| Mortality | 86 (8.0) | 2023 (11.6) | 3222 (18.5) | 5331 (14.8) | <0.001 |
| Chronic ischemic heart failure (% of age group) | 1716 (2.8) | 24048 (12.6) | 15030 (19.5) | 40794 (12.4) | <0.001 |
| Mortality | 53 (3.1) | 1415 (5.9) | 1969 (13.1) | 3437 (8.4) | <0.001 |
| Arrhythmias (% of age group) | 1358 (2.2) | 28839 (15.2) | 24331 (31.5) | 54508 (16.6) | <0.001 |
| Mortality | 64 (4.7) | 2317 (8.0) | 3743 (15.4) | 6124 (11.2) | <0.001 |
| Hypertension | 16979 (28.0) | 110189 (57.9) | 52021 (67.3) | 179189 (54.6) | <0.001 |
| Mortality | 221 (1.3) | 3697 (3.4) | 4836 (9.3) | 8754 (4.9) | <0.001 |
| Chronic pulmonary disease (% of age group) | 2586 (4.3) | 15695 (8.3) | 7255 (9.4) | 25536 (7.8) | <0.001 |
| Mortality | 47 (1.8) | 1019 (6.5) | 1038 (14.3) | 2104 (4.9) | <0.001 |
| Chronic kidney failure (% of age group) | 1463 (2.4) | 20866 (11.0) | 19163 (24.8) | 41492 (12.6) | <0.001 |
| Mortality | 58 (4.0) | 1577 (7.6) | 2746 (14.3) | 4381 (10.6) | <0.001 |
| Diabetes mellitus (% of age group) | 5152 (8.5) | 44188 (23.2) | 19339 (25.0) | 68679 (20.9) | <0.001 |
| Mortality | 113 (2.2) | 2156 (4.9) | 2252 (11.6) | 4521 (6.6) | <0.001 |
| Cachexia/malnutrition (% of age group) | 3107 (5.1) | 11879 (6.2) | 6468 (8.4) | 21454 (6.5) | <0.001 |
| Mortality | 77 (2.5) | 832 (7.0) | 752 (11.6) | 1661 (7.7) | <0.001 |
| Obesity (% of age group) | 6268 (10.3) | 20394 (10.7) | 4658 (6.0) | 31320 (9.5) | <0.001 |
| Mortality | 102 (1.6) | 718 (3.5) | 434 (9.3) | 1254 (4.0) | <0.001 |
| Dementia (% of age group) | 31 (0.1) | 3347 (1.8) | 6248 (8.1) | 9626 (2.9) | <0.001 |
| Mortality | md | 387 (11.6) | 1093 (17.5) | 1482 (15.4) | <0.001* |

+ non-parametric test for trend, if no indication Chi2 test. X due to data protection legislation no data provided. * omitting the group with missing data. md: missing data

| **Supp. table 3 Failure to rescue** | | | | | |
| --- | --- | --- | --- | --- | --- |
|  | **Age group** | | | | |
|  | **<60** | **60-79** | **≥80** | **total** | ***P*‡** |
| Complications (cumulative) | 14309 (23.6) | 55658 (29.2) | 28631 (37.1) | 98598 (30.0) | <0.001 |
| Mortality | 591 (4.1) | 6318 (11.4) | 6790 (23.7) | 13699 (13.9) | <0.001+ |
| Surgical complications | 12865 (21.2) | 45471 (23.9) | 20709 (26.8) | 79045 (24.1) | <0.001 |
| Mortality | 484 (3.8) | 5016 (11.0) | 4930 (23.8) | 10430 (13.2) | <0.001+ |
| Medical complications | 4115 (6.8) | 25526 (13.4) | 16663 (21.6) | 46304 (14.1) | <0.001 |
| Mortality | 515 (12.5) | 5463 (21.4) | 5641 (33.9) | 11619 (25.1) | <0.001+ |
| Ventilation ≥48h | 1594 (2.6) | 9591 (5.0) | 5122 (6.6) | 16307 (5.0) | <0.001 |
| Mortality | 293 (18.4) | 3113 (32.5) | 2687 (52.5) | 6093 (37.4) | <0.001+ |
| CPR | 248 (0.4) | 2158 (1.1) | 1539 2.0) | 3945 (1.2) | <0.001 |
| Mortality | 131 (52.8) | 1341 (62.1) | 1100 (71.5) | 2572 (65.2) | <0.001+ |
| Pulmonary embolism | 498 (0.8) | 2192 (1.2) | 1062 (1.4) | 3752 (1.1) | <0.001 |
| Mortality | 61 (12.3) | 463 (21.1) | 307 (28.9) | 831 (22.2) | <0.001+ |
| Hospital acquired pneumonia | 1223 (2.0) | 7448 (3.9) | 5126 (6.6) | 13797 (4.2) | <0.001 |
| Mortality | 127 (10.4) | 1463 (19.6) | 1601 (31.2) | 3191 (23.1) | <0.001+ |
| Acute kidney injury | 1631 (2.7) | 11547 (6.1) | 7628 (9.9) | 20806(6.3) | <0.001 |
| Mortality | 279 (17.1) | 3117 (27.0) | 3032 (39.8) | 6428 (30.9) | <0.001+ |
| CRRT | 237 (0.4) | 1743 (0.9) | 816 (1.1) | 2796 (0.9) | <0.001 |
| Mortality | 107 (45.2) | 1058 (60.7) | 608 (74.5) | 1773 (63.4) | <0.001+ |
| Clostroides difficile | 248 (0.4) | 1784 (0.9) | 1505 (2.0) | 3537 (1.1) | <0.001 |
| Mortality | 10 (4.0) | 163 (9.1) | 240 (16.0) | 413 (11.7) | <0.001+ |
| Myocardial infarction | 108 (0.2) | 1407 (0.7) | 1090 (1.4) | 2605 (0.8) | <0.001 |
| Mortality | 18 (16.7) | 326 (23.2) | 363 (33.3) | 707 (27.1) | <0.001+ |
| Stroke | 61 (0.1) | 785 (0.4) | 565 (0.7) | 1411 (0.4) | <0.001 |
| Mortality | 11 (18.0) | 159 (20.3) | 171 (30.3) | 341 (242) | <0.001+ |
| Mass transfusion (≥6 units) | 1713 (2.8) | 9018 (4.7) | 4737 (6.1) | 15468 (4.7) | <0.001 |
| Mortality | 248 (14.5) | 2179 (24.2) | 1585 (33.5) | 4012 (25.9) | <0.001+ |
| Relaparotomy | 3267 (5.4) | 11961 (6.3) | 4849 (6.3) | 20077 (6.1) | <0.001 |
| Mortality | 182 (5.6) | 1952 (16.3) | 1469 (30.3) | 3603 (18.0) | <0.001+ |
| Peritonitis | 6266 (10.3) | 22140 (11.6) | 10623 (13.7) | 39029 (11.9) | <0.001 |
| Mortality | 393 (6.3) | 3924 (17.7) | 3717 (35.0) | 8034 (20.6) | <0.001+ |
| Anastomotic leakage | 4102 (6.8) | 12672 (6.7) | 4550 (5.8) | 21324 (6.5) | <0.001 |
| Mortality | 156 (3.8) | 1639 (12.9) | 1361 (29.9) | 3156 (14.8) | <0.001+ |
| Surgical site infection | 4095 (6.8) | 14789 (7.8) | 5769 (7.5) | 24653 (7.5) | <0.001 |
| Mortality | 87 (2.1) | 933 (6.3) | 769 (13.3) | 1789 (7.3) | <0.001+ |
| Bleeding | 1996 (3.3) | 7549 (4.0) | 3518 (4.6) | 13063 (4.0) | <0.001 |
| Mortality | 78 (3.9) | 632 (8.4) | 571 (16.2) | 1281 (9.8) | <0.001+ |

+ non-parametric test for trend, if no indication Chi2 test. CPR: cardio-pulmonary resuscitation; CRRT: Continuous renal replacement therapy. Medical complications: prolonged ventilation, CPR, pulmonary embolism, hospital acquired pneumonia, acute kidney injury, CVVHDF, clostroides infection, myocardial infarction, stroke; Surgical complications: massive transfusion, relaparotomy, peritonitis, anastomotic leakage, surgical site infection and bleeding.

| **Supp. table 4 Crude odds ratios to determine factors influencing failure to rescue** | | |
| --- | --- | --- |
|  | **Crude odds ratio** | ***P*** |
| **Age** |  |  |
| <60 years | 1,0 |  |
| 50-79 years | 3.49 [3.21-3.80] | <0.001 |
| ≥80 years | 9.79 [8.99-10.66] | <0.001 |
| **Sex** |  |  |
| F | 1,0 |  |
| M | 1.23 [1.19-1.27] | <0.001 |
| **Caseload quintile** |  |  |
| Very low | 1,0 |  |
| Low | 0.88 [0.84-0.93] | <0.001 |
| Medium | 0.75 [0.71-0.79] | <0.001 |
| High | 0.70 [0.66-0.74] | <0.001 |
| Very high | 0.63 [0.60-0.66] | <0.001 |
| **Location** |  |  |
| Colon cancer | 1,0 |  |
| Rectum cancer | 0.66 [0.64-0.69] | <0.001 |
| **Admission** |  |  |
| Elective | 1 |  |
| Emergency | 2.34 [2.26-2.42] | <0.001 |

| **Supp. table 5 Logistic regression analysis of failure to rescue by age, including hospital as random effect** | | |
| --- | --- | --- |
|  | **Adjusted odds ratio [95% CI]** | ***P*** |
| **Age** |  |  |
| <60 years | 1,0 |  |
| 60-79 years | 2.23 [2.03-2.45] | <0.001 |
| ≥80 years | 4.11 [3.75-4.12] | <0.001 |
| **Sex** |  |  |
| F | 1,0 |  |
| M | 1.07 [1.03-1.12] | 0.001 |
| **Comorbidity** | 1.27 [1.27-1.28] | <0.001 |
| **Caseload quintile** | 0.95 [0.93-0.98] | <0.001 |
| **Location** |  |  |
| Colon cancer | 1,0 |  |
| Rectum cancer | 0.92 [0.88-0.96] | <0.001 |
| **Admission** |  |  |
| Elective |  |  |
| Emergency | 1.43 [1.37-1.49] | <0.001 |
